# A Lactobacillus-Deficient Vaginal Microbiota Dominates Postpartum Women in Rural Malawi

**DOI:** 10.1128/AEM.02150-17

**Published:** 2018-03-01

**Authors:** Ronan Doyle, Austridia Gondwe, Yue-Mei Fan, Kenneth Maleta, Per Ashorn, Nigel Klein, Kathryn Harris

**Affiliations:** a Institute of Child Health, University College London, London, United Kingdom; b Department for International Health, University of Tampere School of Medicine, Tampere, Finland; c Center for Child Health Research, University of Tampere School of Medicine and Tampere University Hospital, Tampere, Finland; d Department of Pathology, University of Malawi College of Medicine, Blantyre, Malawi; e Department of Paediatrics, University of Tampere School of Medicine, Tampere, Finland; f Department of Microbiology, Virology and Infection Control, Great Ormond Street Hospital NHS Foundation Trust, London, United Kingdom; The Pennsylvania State University

**Keywords:** 16S rRNA gene, Gardnerella vaginalis, Lactobacillus spp., postpartum, sub-Saharan Africa, vaginal microbiota

## Abstract

The bacterial community found in the vagina is an important determinant of a woman's health and disease status. A healthy vaginal microbiota is associated with low species richness and a high proportion of one of a number of different Lactobacillus spp. When disrupted, the resulting abnormal vaginal microbiota is associated with a number of disease states and poor pregnancy outcomes. Studies up until now have concentrated on relatively small numbers of American and European populations that may not capture the full complexity of the community or adequately predict what constitutes a healthy microbiota in all populations. In this study, we sampled and characterized the vaginal microbiota found on vaginal swabs taken postpartum from a cohort of 1,107 women in rural Malawi. We found a population dominated by Gardnerella vaginalis and devoid of the most common vaginal Lactobacillus species, even if the vagina was sampled over a year postpartum. This Lactobacillus-deficient anaerobic community, commonly labeled community state type (CST) 4, could be subdivided into four further communities. A Lactobacillus iners-dominated vaginal microbiota became more common the longer after delivery the vagina was sampled, but G. vaginalis remained the dominant organism. These results outline the difficulty in all-encompassing definitions of what a healthy or abnormal postpartum vaginal microbiota is. Previous identification of community state types and associations among bacterial species, bacterial vaginosis, and adverse birth outcomes may not represent the complex heterogeneity of the microbiota present. (This study has been registered at ClinicalTrials.gov as NCT01239693.)

**IMPORTANCE** A bacterial community in the vaginal tract is dominated by a small number of Lactobacillus species, and when not present there is an increased incidence of inflammatory conditions and adverse birth outcomes. A switch to a vaginal bacterial community lacking in Lactobacillus species is common after pregnancy. In this study, we characterized the postpartum vaginal bacterial community of a large group of women from a resource-poor, undersampled population in rural Malawi. The majority of women were found to have a Lactobacillus-deficient community, and even when sampled a year after delivery the majority of women still did not have Lactobacillus present in their vaginal microbiota. The effect of becoming pregnant again for those who do not revert to a Lactobacillus-dominant community is unknown, and this could suggest that not all Lactobacillus-deficient community structures are adverse. A better understanding of this complex community state type is needed.

## INTRODUCTION

Healthy vaginal community states have been defined as being dominated by a number of different Lactobacillus species (1). Previous studies have found that individuals lacking an abundance of lactobacilli are more likely to develop other conditions, such as bacterial vaginosis (BV) or aerobic vaginitis (AV), leading to an increased risk in transmission of sexually transmitted infections (2) and, if pregnant, premature delivery (3). BV is defined by an overabundance of anaerobic organisms, such as Gardnerella vaginalis, Prevotella spp., and Bacteroides spp. (4), and has previously been identified by a change in vaginal pH, vaginal discharge, and a fishy odor. AV is defined by a Lactobacillus species-deficient community and an overabundance of aerobic bacteria, such as Escherichia coli, Staphylococcus spp., and group B Streptococcus (GBS) spp. (5).

The incidence of a vaginal microbiota lacking Lactobacillus spp. during pregnancy has been associated with adverse birth outcomes, in particular that of preterm birth (6–10). Vaginal microbiota can also undergo extensive shifts postpartum (3, 11). Incidence of preterm birth is highest in low-income countries, such as Malawi (12), and while there are many risk factors involved, infection has been estimated as the probable cause of preterm birth in 25 to 40% of cases, with ascending infection from the vagina through the cervix as the potential source of organisms invading maternal and fetal tissues (13). Many of the organisms historically recovered from intrauterine infections are also commonly found in the genital tract (14), and the presence of these organisms postpartum is associated with a higher risk of developing infections (15).

Previous work has used scoring of Gram stains to diagnose BV (16), but recent studies have utilized molecular techniques focusing on the amplification of the 16S rRNA genes to compare with these scores and elucidate multiple community state types (CSTs). These include whether the vaginal microbiota is dominated by Lactobacillus crispatus (CST 1), Lactobacillus gasseri (CST 2), Lactobacillus iners (CST 3), or Lactobacillus jensenii (CST 5). Those communities that are Lactobacillus species-deficient have been grouped as CST 4 (1, 17). There have been comparatively more 16S rRNA gene high-throughput vaginal microbiota studies focusing on Caucasian cohorts in the United States and Europe than studies focusing on sub-Saharan African populations. The findings from these studies may not be immediately translatable, given the evidence that ethnicity influences the composition of the vaginal microbiota (18). While high estimates of the prevalence of BV in Malawi have been made (19), no attempt so far has been made to characterize the majority of the bacteria involved.

The aim of this study was to characterize the vaginal microbiota of a large cohort of women in rural Malawi using 16S rRNA gene sequencing. We wanted to expand the current knowledge base for the community structure of the vaginal microbiota in a low-income African setting and whether this structure alters over time postpartum. We also wanted to study the correlation between vaginal microbiota and birth outcomes that continues postpartum.

## RESULTS

### Sample collection.

**FIG 1:**
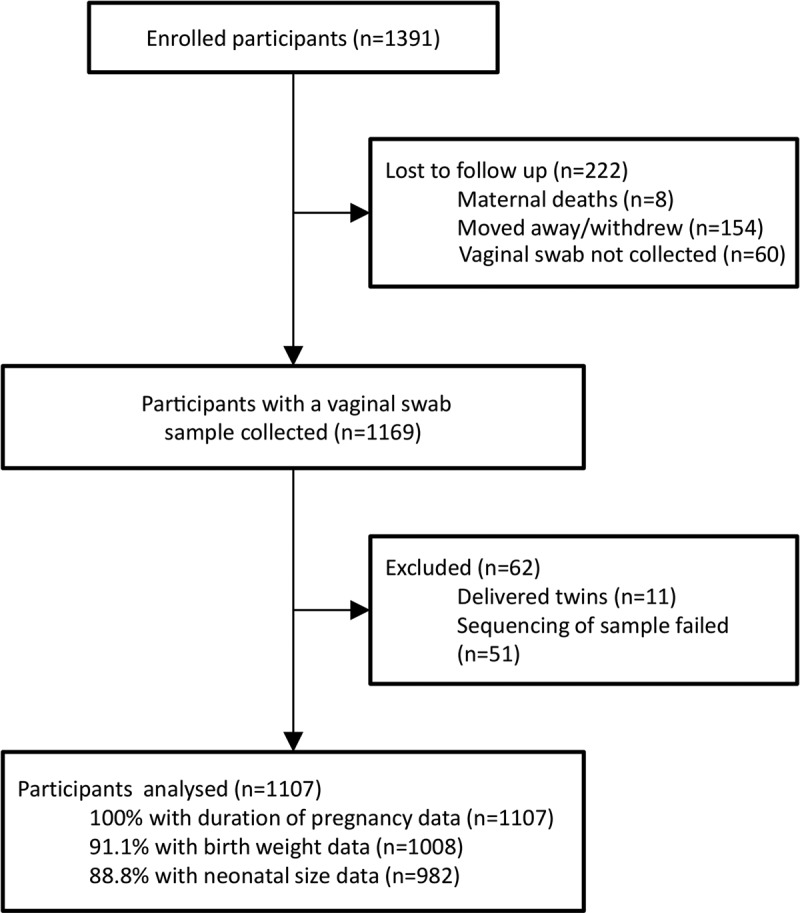
Study participant flow diagram.

Starting in February 2011, a total of 1,391 pregnant women were recruited into the iLiNS-DYAD-M trial, with the last delivery taking place in February 2013. A single vaginal mucus swab sample was taken from each participant approximately 1 week postpartum, and a vaginal microbiota was characterized in 1,107 (79.6%) participants. Of those excluded, 222 were lost to follow-up, 11 were excluded due to twin deliveries, and 51 failed sequencing (Fig. 1). Participants who were included in this substudy were older (25 years versus 24 years; *P* = 0.025), had completed less time in education (3.9 years versus 4.5 years; *P* = 0.049), had a lower proxy for socioeconomic status (37) (−0.05 versus 0.30; *P* < 0.001), and were more likely to be primiparous (first pregnancy; 29.6% versus 20.1%; *P* = 0.001) than those excluded from the analyses (Table 1). We sequenced 1,107 samples from individual participants, and filtering for quality and error allowed analysis at a median depth of 14,585 reads per sample (interquartile range [IQR], 7,986 to 21,659) from 18,661,136 reads across the entire cohort. After sequence filtering steps, 994 participant samples had sufficient sequence depth to be taken forward for analysis.

**TABLE 1:** Baseline characteristics of the included and excluded participants (*n* = 1,391)

Characteristic^*a*^	Included (*n* = 1,107)	Excluded (*n* = 284)	*P* value^*b*^
Mean (SD) BMI (kg/m^2^)	22.1 (2.8)	22.4 (2.9)	0.126
Mean (SD) maternal age (yrs)	25.1 (6.1)	24.2 (6.6)	0.025
Mean (SD) maternal education, completed years of school (yrs)	3.9 (3.4)	4.5 (3.7)	0.049
Mean (SD) socioeconomic score	−0.05 (0.9)	0.30 (1.1)	<0.001
Proportion of primiparous women (%)	29.6	20.1	0.001
Proportion of women with a low BMI (%)^*c*^	4.1	5.7	0.367
Proportion of women with a positive HIV test (%)	12.2	13.9	0.591
Proportion of women with a positive malaria test (RDT) (%)	22.4	23.5	0.749

a SD, standard deviation; BMI, body-mass index; RDT, rapid diagnostic test.

b *P* value obtained from ANOVA (comparison of means) or Fisher's exact test (comparison of proportions).

c A low BMI was defined as <18.5 kg/m^2^.

### The vaginal microbiota of rural Malawian women after pregnancy is dominated by Gardnerella vaginalis, with limited numbers of Lactobacillus spp.

**FIG 2:**
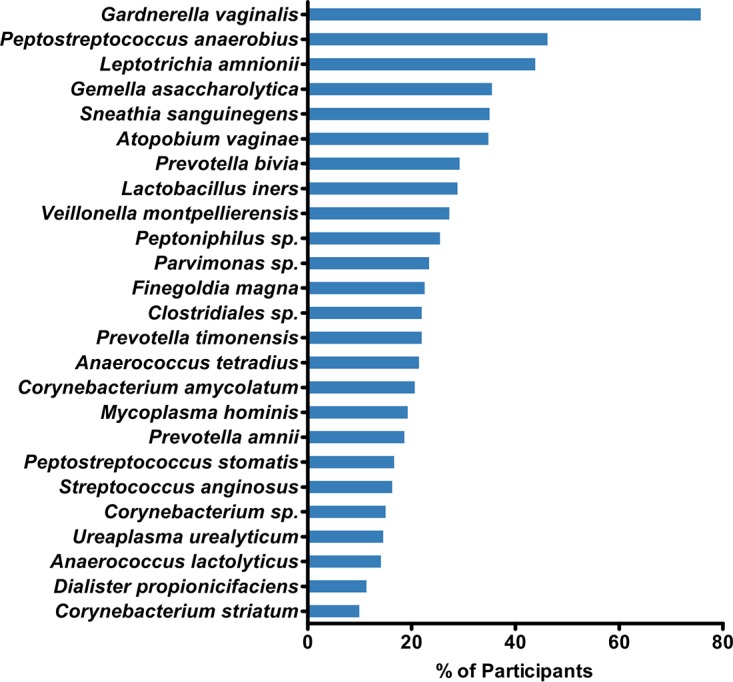
The 25 most prevalent OTUs recovered from participants' vaginal swab samples (*n* = 994).

We found that Gardnerella vaginalis was the most common organism recovered from participants' vaginal swabs (Fig. 2). It was prevalent in 75.7% (752/994) of participants and was the only operational taxonomic unit (OTU) found in more than 50% of individuals. In contrast, Lactobacillus spp. were found in the vaginal microbiota of just 27.1% (269/994) of participants. When lactobacilli were present they were characterized by sequence identity as almost universally Lactobacillus iners, with low prevalence and proportions of Lactobacillus crispatus. Neither Lactobacillus gasseri nor Lactobacillus jensenii was identified in any sample.

### Identification of subtypes in community state type 4.

**FIG 3:**
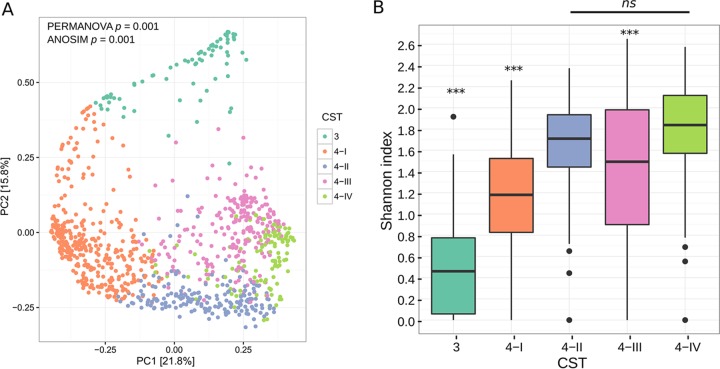
(A) Principal-coordinate analysis of Bray-Curtis distances comparing presence of OTUs in participants' vaginal swab samples. Participants are colored based on *de novo* clustering by the shared presence of OTUs (*n* = 994). (B) Box-and-whisker plot of the distribution of OTU richness. OTU richness was measured among all five CSTs using the Shannon index. Three asterisks (***) above a CST indicate that the difference between it and all other groups was statistically significant (*P* < 0.0001) as determined by Student's *t* test (false discovery rate [FDR]-corrected), and *ns* indicates there was no statistically significant difference between the Shannon index values in CST 4-II and CST-IV.

To elucidate possible similarities in vaginal microbiota community structure between groups of participants, we calculated Bray-Curtis distances across the entire population. We carried out *de novo* clustering on 994 individuals based on the similarity of their microbiota and found that participants separated into five community states (Fig. 3A).

**FIG 4:**
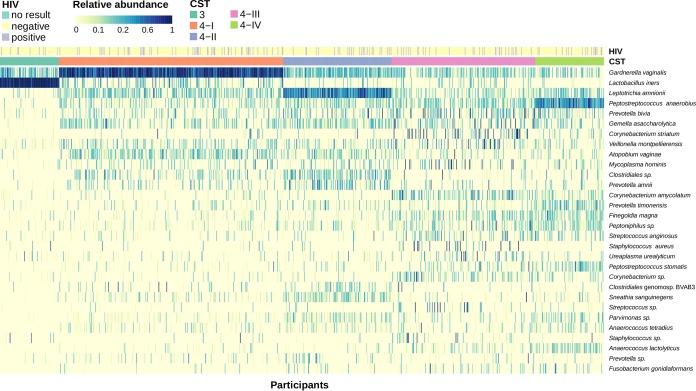
Heat map of the relative abundances of 30 OTUs ordered by CST. Each individual is annotated both by the given CST and by HIV status where information was available (*n* = 994).

The vaginal microbiota of one cluster of participants dominated by Lactobacillus iners was found to be similar to community state type 3 (CST 3) in previous studies (Fig. 4). This was also confirmed when comparing the CST 3 Shannon index to those of the other groups, with CST 3 having a statistically significantly lower Shannon index than all of the CST 4 groups (Fig. 3B). The majority of the other participants had a lactobacillus-deficient community that was instead dominated by bacteria similar to those in the previously studied CST 4. In this study, CST 4 clustered into three more subgroups that were defined by having a high relative abundance of Gardnerella vaginalis (CST 4-I), Leptotrichia amnionii (CST 4-II), or Peptostreptococcus anaerobius (CST 4-IV) or as another group that was not dominated by a single OTU but instead had high abundances of Prevotella spp., Gemella spp., and Corynebacterium spp. (CST 4-III) (Fig. 4). Although there was some crossover, these clusters were confirmed as statistically significantly different from each other by both permutational multivariate analysis of variance (PERMANOVA) (*F* statistic = 159.7; *P* < 0.001) and analysis of similarity (ANOSIM) (*R* = 0.68; *P* < 0.001) tests (Fig. 3A). Both CST 4-I and 4-II also shared many of the same minor OTUs in their communities, such as Gemella asaccharolytica, Prevotella bivia, Veillonella montpellierensis, Atopobium vaginae, Clostridiales sp., and Prevotella amnii. In addition, CST 4-III and 4-IV both had minor OTUs present that were not found in the majority of CST 4-I and 4-II samples, such as Corynebacterium amycolatum, Finegoldia magna, Peptoniphilus sp., Staphylococcus aureus, and Peptostreptococcus stomatis. All the CST 4 subtypes had alpha diversities significantly different from one another, apart from the two groups with the highest Shannon index values, CST 4-II and CST 4-IV (Fig. 3B). CST 4-II had the lowest Shannon index value of the CST 4 subtypes, with a population more likely to consist entirely of Gardnerella vaginalis. Interestingly, we found that although CST 4-III was not dominated by a single OTU like the other groups, it still had a lower Shannon index value than both CST 4-II and CST 4-IV.

### Vaginal microbiota composition varies based on time after delivery.

**FIG 5:**
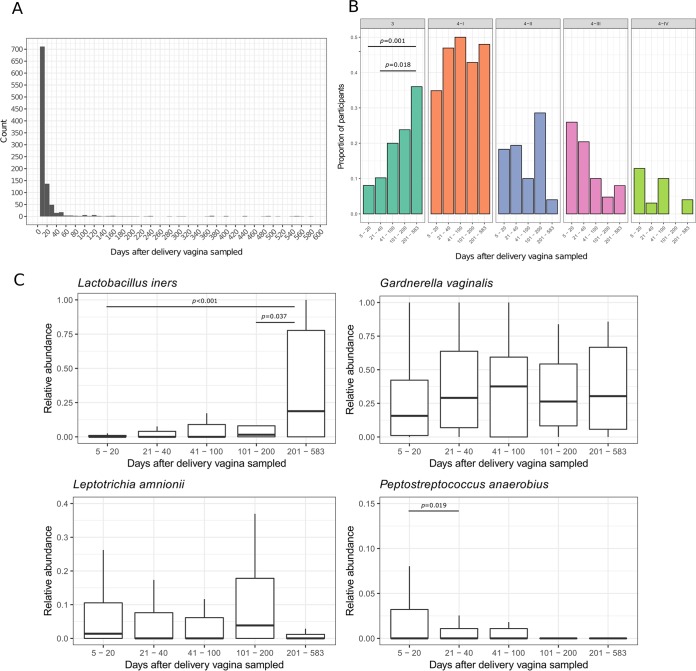
(A) Histogram of the number of days after delivery that vaginal samples were collected. (B) The proportion of participants assigned to each CST stratified by how long after delivery samples were taken. Participants were binned into 5 groups: 5 to 20 days (*n* = 809), 21 to 40 days (*n* = 98), 41 to 100 days (*n* = 40), 101 to 200 days (*n* = 21), and 201 to 583 days (*n* = 26). *P* values show where there was a significant pairwise comparison between groups as determined by Fisher's exact test (FDR-corrected). (C) Box-and-whisker plot showing the association between relative abundance of L. iners, G. vaginalis, L. amnionii, and P. anaerobius and how long after delivery the vagina was sampled. *P* values show where there was a significant pairwise comparison between groups as determined by Student's *t* test (FDR-corrected).

The majority of samples collected in this study were taken from participants less than 20 days after delivery, and some were sampled long after delivery (full range, 5 to 583 days; Fig. 5A). To visualize the possible association between time postpartum and the microbiota present, we binned participants based on how long after delivery they were sampled (Fig. 5B). The proportion of participants defined as CST 3 was low in those sampled less than 20 days after delivery, but CST 3 was the second most abundant CST in samples collected more than 200 days after delivery. This corresponded with a relative decrease in the proportion of participants defined as CST 4-II, 4-III, and 4-IV in samples collected more than 200 days after delivery. We also compared the relative abundances of L. iners in participants grouped by how many days after delivery the sample was taken, and as expected, found relative abundance increased as more participants were defined as CST 3. The two other CST-defining bacteria, L. amnionii and P. anaerobius, were both found to decrease in abundance over time and were almost absent from samples taken more than 200 days after delivery (Fig. 5C). Bacteria associated with CST 4-I remained the most dominant community members in all participants, regardless of the time after delivery the vagina was sampled, and G. vaginalis remained the dominant organism seen in the vaginal microbiota even over a year postpartum (Fig. 5C).

### Other factors influencing vaginal microbial composition.

A total of 13.9% (138/994) of individuals in this study were HIV positive. We annotated the heat map in Fig. 4 with an individual's HIV status to compare the possible association of HIV status with microbial composition, and we also tried to determine whether HIV status correlated with the clusters generated using the Bray-Curtis dissimilarity matrix (see Fig. S1 in the supplemental material). HIV infection did not explain the observed structure of vaginal OTUs among participants. However, differential abundances between OTUs in HIV-negative and HIV-positive participants identified using log-binomial regression showed that the two greatest differences in OTU abundance were in Mycoplasma hominis and BVAB3, with log-2-fold increases in abundance of 0.77 and 0.58 in HIV-positive and HIV-negative participants, respectively. Neither of these associations was statistically significant after adjusting for false discovery rate (FDR) (*q* value < 0.05).

We built ordination plots for other variables that we thought might have an influence on a woman's community composition. We compared participant age, body mass index (BMI), anemic status, presence of malarial infection, if the woman had recently delivered by C-section or vaginally, and geographic location (see Fig. S1 in the supplemental material). The sequencing runs were also compared to see whether processing certain samples together was causing similar profiles of contamination. None of these variables explained the majority of differences between CSTs.

### Association between adverse birth outcomes and the vaginal microbiota soon after delivery.

We wanted to test whether an association between vaginal microbiota and duration of pregnancy, birth weight, newborn length-for-age Z score (LAZ), and newborn head-circumference-for-age Z score (HCZ) remained postpartum. Due to the expected effect of the time after delivery the vagina was sampled, we stratified participants between those sampled on and before 20 days after delivery and those sampled after 20 days. Participants sampled less than 20 days after delivery and categorized as CST 4-IV had the lowest mean duration of pregnancy, lowest mean birth weight, lowest mean LAZ, and lowest mean HCZ. There was a statistically significantly lower mean duration of pregnancy and a statistically significantly lower mean LAZ for participants with vaginal microbiota categorized as CST 4-IV (Table 2). These associations were not seen in participants sampled more than 20 days postpartum (Table 2).

**TABLE 2:** Outcomes by community state type and stratified by whether the sample was collected before 20 days postpartum

Outcome	*n*	Mean (SD) value according to CST					*P* value^*a*^
		3	4-I	4-II	4-III	4-IV	
Samples collected ≤20 days postpartum							
Duration of pregnancy (wks)	809	39.9 (1.7)	39.5 (1.8)	39.7 (1.5)	39.3 (1.8)	38.9 (2.6)	0.001
Birth wt (g)	762	3,092 (527)	2,984 (445)	2,967 (357)	3,011 (429)	2,916 (457)	0.154
Newborn length-for-age Z score	783	−0.8 (1.2)	−0.9 (1.1)	−1.0 (0.9)	−0.9 (1.1)	−1.3 (1.2)	0.037
Newborn head-circumference-for-age Z score	785	−0.0 (1.3)	−0.1 (1.1)	−0.1 (0.9)	−0.0 (1.0)	−0.3 (1.3)	0.246
Samples collected >20 days postpartum							
Duration of pregnancy (wks)	184	38.1 (3.4)	37.6 (5.2)	39.2 (2.5)	37.7 (5.9)	39.5 (2.1)	0.361
Birth wt (g)	141	2,740 (586)	2,931 (526)	2,800 (298)	2,914 (509)	2,870 (237)	0.483
Newborn length-for-age Z score	92	−0.9 (0.9)	−1.4 (1.3)	−1.5 (1.1)	−0.8 (0.9)	−1.9 (1.4)	0.251
Newborn head-circumference-for-age Z score	93	−0.4 (0.8)	−0.5 (1.2)	−0.2 (1.1)	−0.4 (0.9)	−0.1 (0.4)	0.903

a *P* values were calculated by linear regression. Models were adjusted for nutritional intervention, maternal BMI at enrollment, maternal age, proxy for socioeconomic status, number of previous pregnancies, maternal anemia at enrollment, and number of days after delivery that vaginal samples were collected.

## DISCUSSION

In this study, we have characterized the vaginal microbiota of 994 women in southern Africa, which represents the largest cross-sectional study of its kind to date and one of the few studies in an African population (20–22). We have shown that a diverse microbial community deficient in Lactobacillus species dominated the vaginal samples, that these communities clustered into four subgroups, and that a Lactobacillus-deficient microbiota was still the most common community type a year postpartum. An association between higher proportions of P. anaerobius postpartum and a shorter duration of pregnancy and delivery of smaller newborns was seen in those sampled soon after delivery.

The vaginal microbiota was previously represented by five CSTs (1, 3, 23); however, Gajer et al. also previously split CST 4 into two subtypes, 4-A and 4-B (17). CST 4-B was defined by higher abundances of Atopobium spp., Parvimonas spp., Sneathia spp., and Gardnerella spp., which resembled CST 4-I and 4-II in our study. These clustered separately in our study due to differing abundances of Sneathia/Leptotrichia spp. CST 4-A was differentiated based on higher abundances of Peptoniphilus spp., Anaerococcus spp., Corynebacterium spp., and Finegoldia spp. and resembled two subgroups in this study, CST 4-III and 4-IV.

The majority of studies previously undertaken to characterize the vaginal microbiota, especially postpartum, have examined Caucasian populations from the United States or Europe. Ethnicity, geographic location, and lifestyle all seem to contribute to changes in vaginal microbiota, but there is no conclusive evidence as to which is more influential. Regional microbiota differences have previously been established in gut microbiota studies (24); however, comparable data for the vagina from Africa have focused only on those with HIV or diagnosed BV (21, 22). Regional practices such as vaginal douching are common in Malawi (25). However, there is some disagreement on whether this can (26, 27) or cannot (22) alter the vaginal microbiota, and we do not have the required information to make the comparison in this study. Where differences between ethnic populations in the United States were studied, those of an African American ancestry were found to have greater prevalence of Prevotella spp., Dialister spp., Atopobium spp., Gardnerella spp., Peptoniphilus spp., Sneathia sanguinegens, Aerococcus spp., and Finegoldia magna and a decreased prevalence of Lactobacillus spp. compared to members of other ethnic groups without symptoms of BV (1, 18). Black women are on average more likely to be defined as CST 4 than as any other CST (17). Interestingly, many of those genera are found at a greater prevalence across this Malawian cohort than are Lactobacillus spp. In agreement with this study, when a Lactobacillus sp. was found in an African American cohort it was most likely to be L. iners.

Comparisons of results from studies using 16S rRNA gene sequencing to catalogue bacterial species found in vaginal samples can be misleading due to lack of standardization in methodologies used. There are many biases in the preparation of samples and analysis of data that can affect interpretation of the results (28), and comparisons of exact relative abundances between studies with different methods should be avoided. However, we have validated the robustness of our results with mock community data and so can have greater confidence that changes in relative abundances between samples are a reflection of what was in the original sample. The absence of L. crispatus and L. gasseri in our study is interesting, but could possibly be a technical artifact concerning the ability of primers targeting different 16S regions to amplify or differentiate various bacterial species. However, this primer set is used regularly in our clinical diagnostic service, and it has been shown to be capable of detecting these organisms on a frequent basis, so that explanation seems unlikely.

This cross-sectional study sampled the vaginal microbiota at 1 week postpartum. Longitudinal studies have found that during pregnancy specific community structures are more stable than others (17) and that as pregnancy progresses there is an increase in abundance of Lactobacillus spp. (23). However, the postpartum vaginal microbiota has been shown to be capable of an abrupt drop in the abundance of Lactobacillus spp. and an increase in those of Prevotella spp., Anaerococcus spp., Streptococcus spp., and Peptoniphilus spp. (3, 11, 27). One study followed a small cohort for up to a year postpartum and also found the microbiota disturbance to persist (3). An extensive longitudinal study is needed to confirm both the stability of these community structures over time and shifts in microbial composition pre- and postpartum.

Bacterial vaginosis has previously been associated with preterm birth, and we found that the majority of participants in this study were dominated with BV-associated bacteria, similar to BV-associated bacteria found in another large study (23). The effect this may have on subsequent pregnancies is unknown. BV is more prevalent in Malawi than in the United States and Europe, with incidences between 35% and 85.5% reported using a mixture of Amsel and Nugent (based on the observation of specific morphotypes under the microscope after Gram stain) methods (19, 29, 30). CST 4 subgroups in this study contain anaerobic bacteria that could represent an altered BV microbiota in this population; however, we unfortunately do not have Nugent score results to compare associations with symptoms of BV.

Administration of antibiotics in pregnancy in Africa has been shown to reduce the risk of preterm birth and low birth weight, presumably by eradicating certain vaginal bacteria previously associated with bacterial vaginosis and by reducing the risk of ascending intrauterine infection (30, 38). However, the ideal of a healthy community state that encompasses all populations, and an unhealthy state that must be treated, may not exist. Although a Lactobacillus species-dominated microbiota seems to be advantageous, there could be a specific subset of Lactobacillus-deficient communities that is adverse. At the time of publication, it is common when defining vaginal communities to group them into CSTs based on prevalences of certain bacteria. However, we have shown that there are complex bacterial associations that cannot be reliably explained by the original CST definitions. While we have attempted to expand the definitions in this study, it may be the case that a reduction of these data into a few labeled groups cannot be achieved, and new analytical methods that move beyond this should be deployed when analyzing these kinds of data.

## MATERIALS AND METHODS

### Ethics approval and consent to participate.

Written consent was obtained from the mother at enrollment either by signature or by thumbprint if not literate. If a thumbprint was obtained, an impartial witness also attended and signed. This consent procedure and ethical approval were obtained from the College of Medicine Research and Ethics Committee (COMREC), University of Malawi (protocol no. P.08/10/972). The trial was registered at ClinicalTrials.gov as NCT01239693.

### Study design and enrollment.

This cross-sectional study was a substudy of a clinical trial assessing the impact of lipid-based nutrient supplements (LNS) given to mothers during pregnancy and first 6 months of lactation and to children from 6 to 18 months of age on maternal and child health (34). Participants were enrolled prospectively before 20 weeks gestation and followed through pregnancy, childbirth, and beyond. All women enrolled as part of the main trial were accepted as eligible for the presently described substudy.

### Study setting.

Recruitment took place in 4 centers in Mangochi District, Southern Malawi: Mangochi District Hospital, Malindi Hospital, Lungwena Health Center, and Namwera Health Center.

### Collection of birth outcome and baseline data.

At enrollment, participants' weight, height, and hemoglobin concentrations were measured and obstetric history was recorded. Duration of pregnancy was measured using ultrasound. All participants were tested for malarial infection and HIV (unless they were already known to be HIV positive or opted out). At the first home visit to participants 1 to 2 weeks postenrollment, information was gathered on demographic, social, and economic backgrounds. Birth weight was taken as soon as possible after delivery, while newborn length and newborn head circumference were taken at the infant's first clinic visit at 1 to 2 weeks old.

### Sample collection.

Vaginal swabs were collected at the health centers during a postpartum visit that took place within the first 2 weeks of delivery. The full range of collection times was much larger, with some samples collected nearly 2 years postdelivery. A nurse collected the sample by inserting the swab approximately 7 cm deep past the vaginal introitus, rotating it 3 times back and forth, and then removing the swab and placing it back into the tube. If sample collection took place at an outlying health center or Malindi Hospital, the samples were stored at −20°C for a maximum of 2 days before being transferred to −80°C storage at Mangochi District Hospital.

### DNA extraction.

In preparation for extraction of genomic DNA, vaginal swabs were submerged in 200 μl of buffer AE (10 mM Tris-Cl, 0.5 mM EDTA, pH 9.0; Qiagen) for 1 min, and all liquid was expressed out of the swab before removal. Extraction was carried out using the QIAmp DNA minikit (Qiagen) as per the manufacturer's protocol, with an additional bacterial cell wall disruption step after lysis with proteinase K. In the additional step, 0.1-mm glass beads (Lysing Matrix B; MP Biomedicals) were added to each sample and the 2-ml tubes were shaken on a cell disrupter (Vortex Genie 2; Scientific Industries) for 10 min at the highest speed. For every 10 extractions, a negative extraction control was included (200 μl buffer AE).

### 16S rRNA gene amplicon high-throughput sequencing.

Library preparation was carried out on extracted DNA using dual barcoded primers with attached Illumina-compatible adapters targeting the V5 to V7 regions of the 16S rRNA gene (785F, 5′-GGATTAGATACCCBRGTAGTC-3′, and 1175R, 5′-ACGTCRTCCCCDCCTTCCTC-3′) as previously published (35). Each library preparation PCR was carried out with 1× Molzym PCR buffer, 200 μM deoxynucleoside triphosphates (dNTPs) (Bioline), 0.4 μM forward and reverse primers, 25 mM Moltaq, 5 μl template DNA, and molecular grade water (Bioline) to give a final reaction volume of 25 μl. Vaginal 16S rRNA genes were amplified under the following conditions: 94°C for 3 min, followed by 30 cycles of 94°C for 30 s, 60°C for 40 s, and 72°C for 90 s, with a final extension cycle of 72°C for 10 min. The resulting amplicon was cleaned and pooled using SequalPrep normalization plate kits (Invitrogen) and AMPure XP beads (Beckman Coulter) both as per manufacturer's protocol. Each plate was pooled into an equimolar final library after quantification using a Qubit 2.0 fluorometer (Life Technologies). The library was loaded onto a MiSeq instrument (Illumina) as per the manufacturer's protocol for 500-cycle V2 kits, with the addition of custom sequencing primers for read 1 (TACCGGGACTTAGGATTAGATACCCBRGTAGTC), read 2 (AACACGTTTTAACGTCRTCCCCDCCTTCCTC), and index 1 (GAGGAAGGHGGGGAYGACGTTAAAACGTGTT).

### Bioinformatic analysis.

Paired-end sequenced reads from each MiSeq run were merged using FLASH (Fast Length Adjustment of SHort reads) software (36), demultiplexed, and pooled. Sequences were clustered into operational taxonomic units (OTUs) using QIIME v1.8.0 (31) at 97% sequence similarity using closed-reference OTU picking against a small custom database of full-length 16S rRNA gene sequences. Any sequences that failed to match at 97% were assigned against the full Greengenes database. Mock communities of 21 bacterial strains (BEI Resources) were sequenced alongside samples in each sequencing run, and these were used to calculate sensitivity and to provide a cutoff between OTUs considered to be present in the sample and those considered contamination. Based on the recreation of sequenced mock community results we filtered the data set to keep only OTUs that were present at >3 reads in >5% of all samples. Interrun variability was validated as well, using the relative abundances of strains in the mock community samples. Negative library preparation and extraction controls (molecular grade H_2_O; Bioline) were also sequenced, and OTUs present in negative controls after filtering steps were removed from the entire data set. After filtering steps, any samples with less than 1,000 reads were also removed. *De novo* clustering into CSTs was performed on the Bray-Curtis distances between all samples using the partitioning around medoids algorithm in R as previously described (3). Clustering was used to bin participants into groups based on how similar their vaginal microbiota were to each other, with the number of clusters determined from the gap statistic (see Fig. S2 in the supplemental material). Both PERMANOVA and ANOSIM tests were performed through the vegan package (version 2.4) in R using Bray-Curtis dissimilarities with 999 permutations. For the PERMANOVA test, nutritional intervention, maternal BMI at enrollment, maternal age, HIV status, proxy for socioeconomic status, number of previous pregnancies, maternal anemia at enrollment, and number of days after delivery the sample was taken were all added into the model as covariates.

### Statistical analysis.

All regression models comparing OTU abundances and birth outcomes were adjusted for nutritional intervention, maternal BMI at enrollment, maternal age, proxy for socioeconomic status, number of previous pregnancies, maternal anemia at enrollment, and site of enrollment. Multiple comparison *q* values were calculated using the Benjamini-Hochberg correction to control the false discovery rate. Birth weight as measured was used if recorded within 48 h of delivery; if not, birth weight was back calculated from weight measured at 6 or 13 days. If the weight was first measured within 2 to 5 days after delivery, when infants usually lose weight, birth weight was estimated by applying an age-dependent multiplicative factor to the measured weight (32). Length-for-age Z score (LAZ) and head-circumference-for-age Z score (HCZ) were calculated using the WHO Child Growth Standards (33). The proxy for socioeconomic status used in this study was calculated as previously stated (37). Participants were grouped based on how many days after delivery they were sampled, and bin lengths were chosen to try to evenly distribute participants across groups. Statistical analyses were carried out with Stata v13 and R v3.1.0. Adjusted linear regression models were used to compare mean duration of pregnancy, birth weight, LAZ, and HCZ with CST. Covariates entered into adjusted models were entered into the model in a one-step forced entry method.

### Accession number(s).

All sequence data used in this study can be found in the EBI European Nucleotide Archive. The run accession numbers are ERR1554775, ERR1554776, ERR1554777, ERR1554778, ERR1558673, ERR1558674, ERR1558675, and ERR1558676. The study accession number is PRJEB15035. Mapping metadata for all sequencing runs can be found in Table S1 in the supplemental material.

## Supplementary Material

Supplemental material
