# Supplementary material for: A Lactobacillus-Deficient Vaginal Microbiota Dominates Postpartum Women in Rural Malawi
Source: Appl Environ Microbiol. 2018 Mar 1;84(6):e02150-17. doi: 10.1128/AEM.02150-17 (PMC5835753; doi:10.1128/AEM.02150-17)
Supplement: Supplemental material [file supp_84_6_e02150-17__index.html]

Supplemental material 

# A Lactobacillus-Deficient Vaginal Microbiota Dominates Postpartum Women in Rural Malawi

## Supplemental material

- Supplemental file 1 -

  Multiple PCoA plots of Bray-Curtis distances comparing factors potentially influencing the vaginal microbiome (Fig. S1); gap statistic values at different cluster numbers (Fig. S2); legend to Table S1.

  PDF, 975K
- Supplemental file 2 -

  List of samples used in study, respective index sequences, and metadata (Table S1).

  XLSX, 59K
