# Supplementary material for: A Lactobacillus-Deficient Vaginal Microbiota Dominates Postpartum Women in Rural Malawi
Source: Appl Environ Microbiol. 2018 Mar 1;84(6):e02150-17. doi: 10.1128/AEM.02150-17 (PMC5835753; doi:10.1128/AEM.02150-17)

Lactobacillus-deficient vaginal microbiomes dominate post-partum women in rural Malawi

Ronan Doyle, Austridia Gondwe, Yue-Mei Fan, Kenneth Maleta, Per Ashorn, Nigel Klein, Kathryn Harris

Supplementary Materials

Figure S1. Multiple PCoA plots of Bray-Curtis distances comparing potential influencing factors on the vaginal microbiome. Participants are coloured based various categorical and continuous variables that have the potential of influencing community composition (n=994).

Figure S2. Gap statistic values at different cluster numbers (k). Analysis was based on Bray-Curtis distances calculated from 994 individuals.

Table S1. List of samples used in study, respective index sequences and metadata (Table_S1.xlsx).

Figure S1.

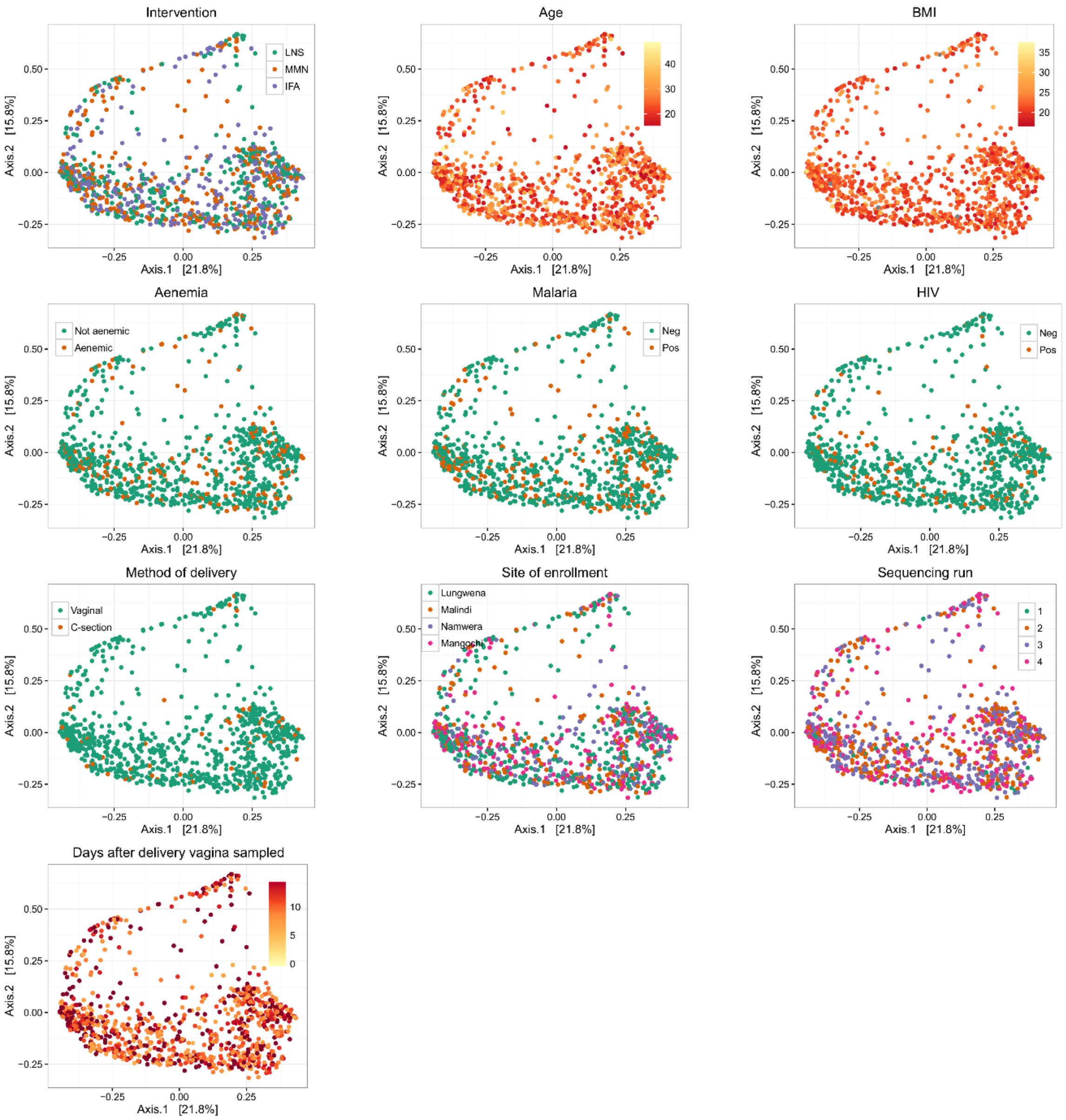

Intervention
LNS
MMN
IFA
Age
BMI
Aenemia
Not aenemic
Aenemic
Malaria
Neg
Pos
HIV
Method of delivery
Vaginal
C-section
Site of enrollment
Lungwena
Malindi
Namwera
Mangochi
Sequencing run
Days after delivery vagina sampled
Axis.1 [21.8%]
Axis.2 [15.8%]

Figure S2.

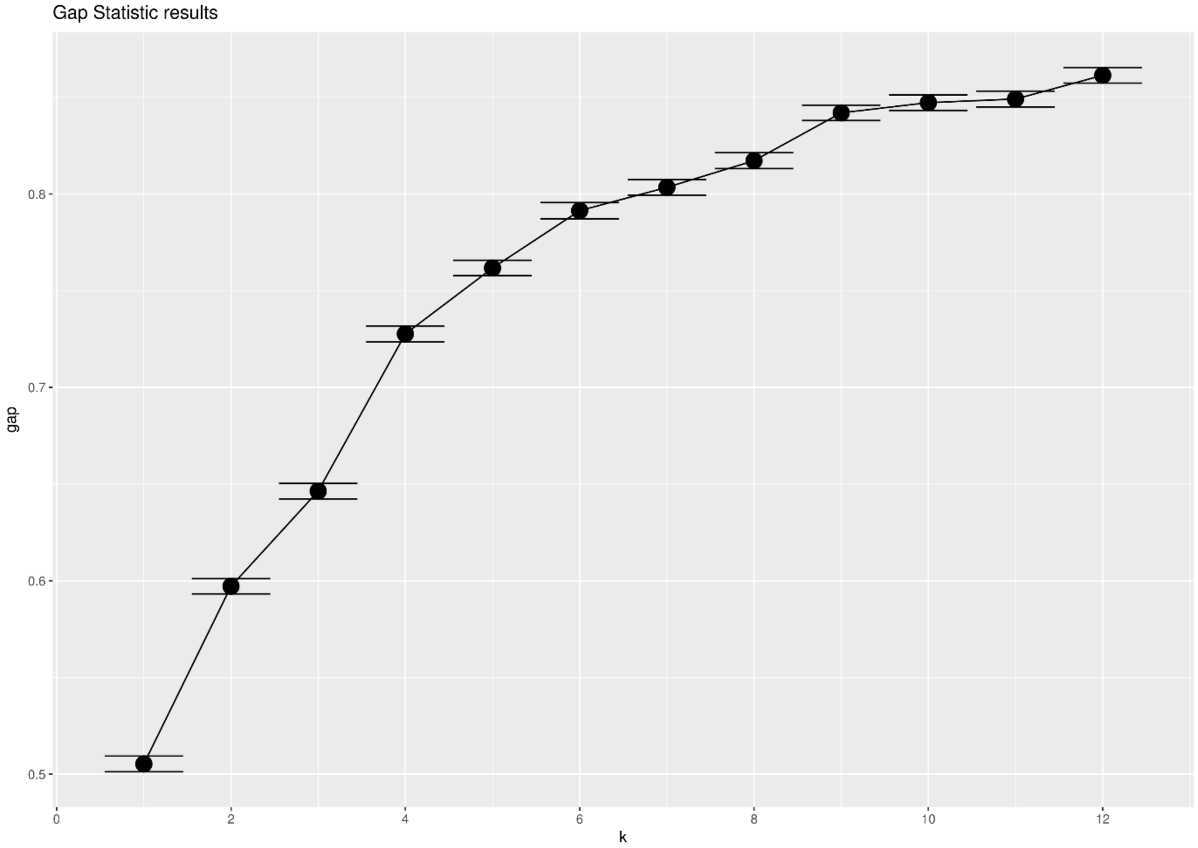

Supplement: Supplemental material [file AEM.02150-17_zam006188376s1.pdf]
